# Differential antioxidant enzyme profiles reveal early molecular signatures of virulence in *Trypanosoma cruzi* DTU-TcI and DTU-TcVI strains

**DOI:** 10.3389/fcimb.2025.1664827

**Published:** 2025-11-28

**Authors:** Edward Valencia Ayala, João Reis Cunha, Maritza Calderón Sánchez, Angela Giovanna Vidal Riva, Daniella Castanheira Bartholomeu, Alexandre Ferreira Marques

**Affiliations:** 1 Laboratorio de Parasitología Molecular y Celular, Facultad de Ciencias Biológicas, Universidad Nacional Mayor de San Marcos, Lima, Peru; 2 Instituto de Investigación - Centro de Investigación en Virología, Facultad de Medicina Humana, Universidad de San Martin de Porres, Lima, Peru; 3 Laboratório de Imunologia e Genômica de Parásitos - Departamento de Parasitologia, Instituto de Ciências Biológicas/ICB, Universidade Federal de Minas Gerais, Minas Gerais, Brazil; 4 Laboratorio de Investigación en Enfermedades Infecciosas - Laboratorio de Biología Molecular, Facultad de Ciencias y Filosofía, Universidad Peruana Cayetano Heredia, Lima, Peru; 5 Center for Molecular and Cellular Biosciences, School of Biological, Environmental, and Earth Sciences, University of Southern Mississippi, Hattiesburg, MS, United States

**Keywords:** *Trypanosoma cruzi*, antioxidant, Chagas disease, oxidative stress, parasite

## Abstract

**Background:**

*Trypanosoma cruzi*, the causative agent of Chagas disease, exhibits significant genetic and phenotypic diversity that influences clinical outcomes. Antioxidant enzymes are crucial for parasite survival under host-induced oxidative stress, yet their role as determinants of virulence remains underexplored.

**Objective:**

This study aimed to characterize and compare early antioxidant responses between the virulent *T. cruzi* CL Brener clone and a non-virulent strain isolated from Arequipa, Peru, to test the hypothesis that elevated antioxidant enzyme expression is associated with parasite virulence.

**Methods:**

A reactivation protocol using *Triatoma infestans* restored infectivity in the non-virulent Arequipa strain. Comparative analyses were performed between the non-virulent Arequipa strain (AQP300), the reactivated strain (AQP-RE), and the CL Brener clone using *in vitro* macrophage infection models, flow cytometry, gene expression profiling, and *in vivo* infection assays in C57BL/6 mice.

**Results:**

Both AQP-RE strain and CL Brener clone established infections in mice, whereas the AQP300 remained non-infective. Genome analysis confirmed the conservation of seven key antioxidant genes across strains. Despite similar phagocytosis rates, macrophages infected with CL Brener exhibited significantly lower nitric oxide and reactive oxygen species production. In contrast, CL Brener triggered robust upregulation of antioxidant genes (TcAPX, TcCPX, TcMPX, TcTR, and TcSODs) at 3- and 24-hours post-infection. *In vivo*, CL Brener induced significantly higher parasitemia, cardiac parasite burden, and sustained proinflammatory cytokine expression (IFN-γ, TNF-α) compared to AQP-RE. Elevated IL-10 expression in AQP-RE-infected mice during early infection suggests enhanced immune regulation in response to lower virulence.

**Conclusions:**

Enhanced early expression of antioxidant enzymes appears to correlate with *T. cruzi* virulence and persistence, suggesting a potential role for these molecules as biomarkers or therapeutic targets. These observations may help clarify strain-specific mechanisms contributing to Chagas disease pathogenesis.

## Introduction

Chagas disease (CD), caused by the protozoan parasite *Trypanosoma cruzi*, remains a major neglected tropical disease in Latin America, affecting an estimated 6–7 million people worldwide (Lidani et al., 2019; Nepomuceno de Andrade et al., 2024). It is estimated that around a million people are infected in the United States (Agudelo Higuita et al., 2024; Bern et al., 2011). Transmission occurs primarily through contact with infected triatomine bugs, and the disease can progress to chronic stages characterized by severe cardiac and gastrointestinal complications (Hochberg and Montgomery, 2023). Despite decades of research, effective vaccines are lacking, and current treatment options remain limited and less effective in chronic infections. *T. cruzi* exhibits remarkable genetic and biological diversity and is classified into seven discrete typing units (DTUs), designated as TcI to TcVI and Tcba (Zingales and Bartholomeu, 2022; Zingales et al., 2012). In Peru, and particularly in the endemic region of Arequipa, TcI predominates in both sylvatic and domestic transmission cycles and is responsible for a high burden of human infections. The domestic vector *Triatoma infestans* continues to drive transmission in the region despite vector control efforts (Bowman et al., 2008; Carbajal-de-la-Fuente et al., 2022).

Upon infection, *T. cruzi* exhibits a broad host-cell tropism and the ability to evade immune responses, resulting in chronic infection in a significant proportion of cases. Macrophages play a significant role in early parasite control by producing reactive oxygen species (ROS) and nitric oxide (NO), which have potent antiparasitic activity (Dias et al., 2017; Vellozo et al., 2023). However, ROS can also have paradoxical effects. In addition to mediating parasite killing, they can modulate host immunity and promote intracellular conditions favorable to *T. cruzi* survival by inducing oxidative stress (Goes et al., 2016; Paiva et al., 2018). To cope with this hostile environment, *T. cruzi* relies on a suite of antioxidant enzymes, including glutathione peroxidases (TcGPX I and II), tryparedoxin peroxidases (TcCPX and TcMPX), an ascorbate peroxidase (TcAPX), and iron-dependent superoxide dismutase (Fe-SODs) (Maldonado et al., 2020; Piacenza et al., 2013; Santi and Murta, 2022). These enzymes are functionally supported by the parasite’s unique trypanothione-based redox system, which includes trypanothione synthetase (TcTrS) and trypanothione reductase (TcTrR) (Battista et al., 2020; Gonzalez-Montero et al., 2024). These antioxidant defenses not only promote parasite persistence but are increasingly recognized as potential virulence factors. For instance, TcCPX can elicit Th1-biased immune responses that may exacerbate host tissue damage (Osorio et al., 2012; Piacenza et al., 2013).

Despite this growing understanding, few studies have examined how antioxidant activity differs among *T. cruzi* strains with distinct virulence phenotypes. Early induction of antioxidant enzymes in *T. cruzi* is increasingly recognized as a determinant of parasite virulence and disease progression. Overexpression of peroxiredoxins, such as TcCPX and TcMPX, enhances resistance to reactive oxygen and nitrogen species *in vitro* and increases parasitemia and tissue inflammation in infected mice (Cardoso et al., 2015). Similar patterns have been reported for trypanothione reductase, where inhibition reduces parasitemia and mortality in animal models (Mendonca et al., 2018). Proteomic analyses have shown that metacyclic trypomastigotes, responsible for establishing mammalian infection, exhibit elevated levels of antioxidant enzymes compared to less infective life stages (Cardoso et al., 2015). Moreover, comparisons across different *T. cruzi* strains demonstrate that more virulent isolates express higher levels of antioxidant enzymes (Piacenza et al., 2013). These observations suggest that early expression of antioxidant enzymes may contribute to virulence and disease outcome. Here, we aimed to characterize and compare the early antioxidant responses of the virulent CL Brener clone and the non-virulent *T. cruzi* strain isolated from Arequipa. We hypothesize that enhanced expression of antioxidant enzymes during the initial phase of infection is a determinant of parasite virulence and represents a potential biomarker or therapeutic target in Chagas disease.

## Materials and methods

### Ethics statement

This study was carried out strictly in accordance with the recommendations in the Guide for the Care and Use of Laboratory Animals of the Brazilian National Council of Animal Experimentation (http://www.cobea.org.br/) and the Federal Law 11.794 (October 8, 2008). The institutional Committee for Animal Ethics of the Federal University of Minas Gerais (License protocol number 92/2009) approved all the procedures used in the present study.

### Parasites

Trypomastigotes of the Arequipa *T. cruzi* strain and the CL Brener clone were cultured in rat L6 myoblast cells using RPMI-1640 medium supplemented with antibiotics (penicillin 60 mg/L and streptomycin 10 mg/L) and 10% fetal bovine serum (FBS), at 37°C in 5% CO_2_ atmosphere (Contreras et al., 1985). The Arequipa strain was donated by Gilman’s research group at the Laboratory of Infectious Diseases Research, Universidad Peruana Cayetano Heredia (UPCH), Peru, and the CL Brener clone was donated by Bartholomeu’s group at the Laboratory of Immunology and Genomics of Parasites, Universidade Federal de Minas Gerais (UFMG), Brazil. Previous experimental infections using the Arequipa strain in mice consistently failed to produce detectable parasitemia, and the strain was thus classified as non-virulent (AQP300). Subsequently, this strain was reactivated (AQP-RE) and used to infect mice and evaluate antioxidant activity in macrophages.

### Reactivation and purification of metacyclic forms

To reactivate the non-virulent strain (AQP300), fifth-instar nymphs of *Triatoma infestans* were artificially fed with blood containing 10^6^ epimastigotes/ml (Kollien and Schaub, 2000). Between days 25 and 28 post-feeding, the contents of the intestinal and rectal ampulla were collected in sterile PBS (pH 7.2, 4°C). The suspension was sterile gas-filtered and subjected to differential centrifugation: 200 g for 5 min to remove macroscopic debris (discard the pellet), and subsequently 3500 g for 10 min at 4°C to recover the parasites (Bonaldo et al., 1988; Contreras et al., 1985). The pellet was resuspended in sterile PBS, and the metacyclic trypomastigotes were quantified in a Neubauer chamber. The percentage of transformation of epimastigotes to metacyclics in triatomines was 35–45% (reported range between 30–50%), and a typical yield of 1 - 5 × 10^6^ metacyclics was obtained per gut group, depending on the initial load and time of collection (Kollien and Schaub, 2000). For *in vivo* experiments, purified metacyclics were adjusted to 10^5^ parasites/mouse in sterile PBS and inoculated i.p. into C57BL/6 mice. Weight, parasitemia, and animal welfare were monitored to continue with the following experiments.

### Purification and immunophenotyping of cells

Peritoneal cells were elicited in C57BL/6 mice by intraperitoneal injection of 2 mL thioglycolate broth per animal. After 72 hours, mice were euthanized by CO_2_ inhalation into an unoccupied and clean chamber, followed by cervical dislocation (Johnson and Patterson-Kane, 2020). Peritoneal cells were harvested using 5 mL of sterile cold PBS containing 1% sodium heparin (5000 IU/mL) (Layoun et al., 2015). Cells were kept on ice and centrifuged at 260 g for 10 minutes at 4 °C. The cell pellet was resuspended in RPMI medium with 10% FBS. Cells were then incubated with fluorochrome-conjugated antibodies for 20 minutes at room temperature, protected from light (**Supplementary Table 1**). After incubation, the cells were centrifuged again at 260 g for 10 minutes and resuspended in 200 μL of PBS for immediate analysis by flow cytometry (Perfetto et al., 2004), using a Cytek Northern Lights (Cytek Biosciences Inc., China). Data were analyzed using FlowJo 10.0 software (Tree Star, Ashland, OR, USA).

### Detection of nitric oxide and reactive oxygen species

Trypomastigotes from CL Brener, AQP300, and AQP-RE strains, along with Zymosan A particles (Saccharomyces cerevisiae; Sigma-Aldrich, was used as a positive control for phagocytosis and ROS/NO production), were labeled with 5 mM carboxyfluorescein succinimidyl ester (CFSE, Invitrogen) for 10 minutes at 37 °C in PBS (Koo et al., 2016) followed by two washes in PBS containing 1% BSA to remove unbound dye. Labeled Zymosan A was resuspended in RPMI and adjusted to 20 mg/mL (stock). Next, peritoneal macrophages (2 x 10^6^ cells/mL) were infected with the labeled trypomastigotes at a 5:1 parasite-to-macrophage ratio or incubated with CFSE-Zymosan A at a 10:1 particle-to-cell ratio (corresponding to ~200 µg/mL final concentration) for 3 hours at 37°C, under gentle orbital agitation (80 rpm) to ensure uniform suspension contact without adherence to plastic surfaces. Non-internalized parasites or excess Zymosan A were removed by washing twice with sterile PBS. Cells were then centrifuged at 260 g for 10 minutes and resuspended in 200 μL of PBS for flow cytometry analysis (Harboe et al., 2012), with 50,000 events acquired within the macrophage gate. For ROS detection, macrophages were treated with 20 μM of carboxy-H_2_DCFDA (5- or 6-carboxy-2’,7’-dichlorodihydrofluorescein diacetate) (Duanghathaipornsuk et al., 2021). For NO detection, macrophages were treated with 2 mM of DAF-2DA (4,5-diaminofluorescein diacetate). To evaluate ROS and NO inhibition, 10 mM diphenyleneiodonium chloride (DPI) and 10 mM aminoguanidine (AG) were used to inhibit NADPH oxidase and iNOS activity, respectively (Dhiman and Garg, 2011).

### Read mapping, assembly, and primer design for antioxidant genes

To investigate genetic variation in antioxidant genes, genomic sequencing reads of the Arequipa strain were retrieved from the NCBI Sequence Read Archive (SRA; accession number PRJNA274442) and compared to the CL Brener reference genome. Quality control of the reads was performed using Trimmomatic, with a minimum Phred score of 20 and read length cutoff of 50 bp (Bolger et al., 2014). Cleaned reads were mapped to the CL Brener non-Esmo-like haplotype (version 20), obtained from the TriTrypDB database, using Bowtie 2 (Langmead and Salzberg, 2012). Reads corresponding to annotated antioxidant genes, *ascorbate peroxidase*, *iron superoxide dismutase*, *trypanothione synthetase*, *trypanothione reductase*, and *tryparedoxin peroxidase*, were extracted and assembled using the CAP3 Contig Assembly Program (Huang and Madan, 1999). Resulting contigs were aligned to the reference genome using BLASTn (Altschul et al., 1990) and refined using the Contig Editor Program (GeneStudio, USA) until scaffolds for each antioxidant gene were obtained (**Supplementary Information**). Highly conserved regions were selected for primer design using EPrimer3 (http://bioinfo.nhri.org.tw/cgi-in/emboss/eprimer3), a tool based on the EMBOSS suite (Rice et al., 2000). These primers (**Supplementary Table 2**) were subsequently used for qPCR analysis of gene expression.

### Gene expression analysis of antioxidant enzymes

Peritoneal macrophages (1×10^6^ cells/mL) were infected with trypomastigotes from the CL Brener, AQP300 and AQP-RE strains at a parasite-to-cell ratio of 5:1. Samples were collected at 3- and 24-hours post-infection. Total RNA was extracted using TRIzol reagent (Invitrogen, USA) following the manufacturer’s instructions. cDNA was synthesized using reverse transcription with oligo(dT) primers and M-MLV reverse transcriptase (Promega, USA) (Green and Sambrook, 2012). Quantitative PCR was performed in 15 μL reactions using SYBR Green Master Mix (Applied Biosystems, USA), 0.1 μM of each primer, and 2 μL of cDNA. The amplification conditions were: 95 °C for 10 min, followed by 40 cycles at 95 °C for 30 s and 60 °C for 1 min. Expression levels were normalized to TcGAPDH (XM_814806) as a constitutive control. Relative expression was calculated using the 2^–ΔCt method (Livak and Schmittgen, 2001). Data acquisition was done on a StepOne Plus Real-Time PCR System (Applied Biosystems-USA).

### Assessment of parasitemia and tissue parasite load in C57BL/6 mice

C57BL/6 female mice (6–8 weeks old) were maintained at the animal facility of the Universidade Federal de Minas Gerais (UFMG), Brazil. All procedures were conducted under protocols approved by the UFMG Animal Ethics Committee (Protocol No. 92/2009). Mice (n=12 per group) were infected with 1×10^4^; blood trypomastigotes of the AQP-RE strain or CL Brener clone suspended in 200 µL sterile phosphate-buffered saline (PBS, pH 7.4). An uninfected control group (n=4) was included. Parasitemia was monitored from days 5 to 30 post-infection via tail vein blood sampling (5 μL), counted in 50 microscopic fields at 400× magnification (Brener, 1965). Parasite load in cardiac tissue was determined by real-time PCR using DNA extracted from 25 mg of homogenized heart tissue via the phenol-chloroform method (Vago et al., 2000). qPCR reactions were prepared using TaqMan 2X Master Mix with uracil-n-glycosylase (Applied Biosystems), 1 μM primers (cruzi1: 5’-ASTCGGCTGATCGTTTTCGA-3’ and cruzi2: 5’-AATTCCTCCAAGCAGCGGATA-3’), and 0.2 μM probe (cruzi3: 5’-CACACACTGGACACCAA-3’, labeled 5’ with FAM (6-carboxyfluorescein) and 3’ with MGB (minor groove binder)) in a 20 μL volume with 100 ng of DNA (Piron et al., 2007). Amplification was performed at 50 °C for 2 min, 95 °C for 10 min, followed by 40 cycles of 95 °C for 15 s, 58 °C for 1 min, and 72 °C for 1 min. Data acquisition was done on a StepOne Plus Real-Time PCR System (Applied Biosystems-USA). Quantitative PCR (qPCR) was performed using a standard curve that was generated from 10-fold serial dilutions of genomic *T. cruzi* DNA (ranging from 10^6 to 10^0 parasite equivalents per reaction) mixed with 100 ng of uninfected mouse DNA to simulate tissue matrix effects. Parasite equivalents were interpolated from the standard curve and expressed as equivalent parasites per mg of tissue (Eq-p/mg) (Cummings and Tarleton, 2003; Duffy et al., 2009; Piron et al., 2007).

### Cytokine expression in cardiac tissue

To evaluate host immune responses, the expression of IFN-γ, TNF-α, and IL-10 in cardiac tissue was assessed via reverse transcription and qPCR, following protocols described above for antioxidant gene expression. Total RNA was extracted from 25 mg of heart tissue using TRIzol. Mouse β-actin (Actb, NM_007393.5) served as the endogenous reference gene. Relative gene expression was calculated using the 2^–ΔCt method (Livak and Schmittgen, 2001). The oligonucleotide sequences used for amplification were as follows: β-actin (BaF, 5′-GCTTCTTTGCAGCTCCTTCGT-3′; BaR, 5′-CGTCATCCATGGCGAACTG-3′), IL-10 (10F, 5′-CATTTGAATTCCCTGGGTGAGA-3′;10R, 5′-TGCTCCACTGCCTTGCTCTT-3′), TNF-α (TFF,5′-CATCTTCTCAAAATTCGAGTGACAA-3′;TFR,5′-CCTCCACTTGGTGGTTTGCT-3′), and INF-γ (IFF, 5′-TCAGCAACAGCAAGGCGAAA-3′; IFR, 5′-CCGCTTCCTGAGGCTGGAT-3′).

### Statistical analysis

The experiments were performed in triplicate with two repeats. In this study, the results were evaluated by the Mann Whitney test for non-parametric data, using the Graph Prism Instat program, with a 95% confidence interval (p <0.05).

## Results

### Validation of the infection model and early macrophage response

**Figure 1:**
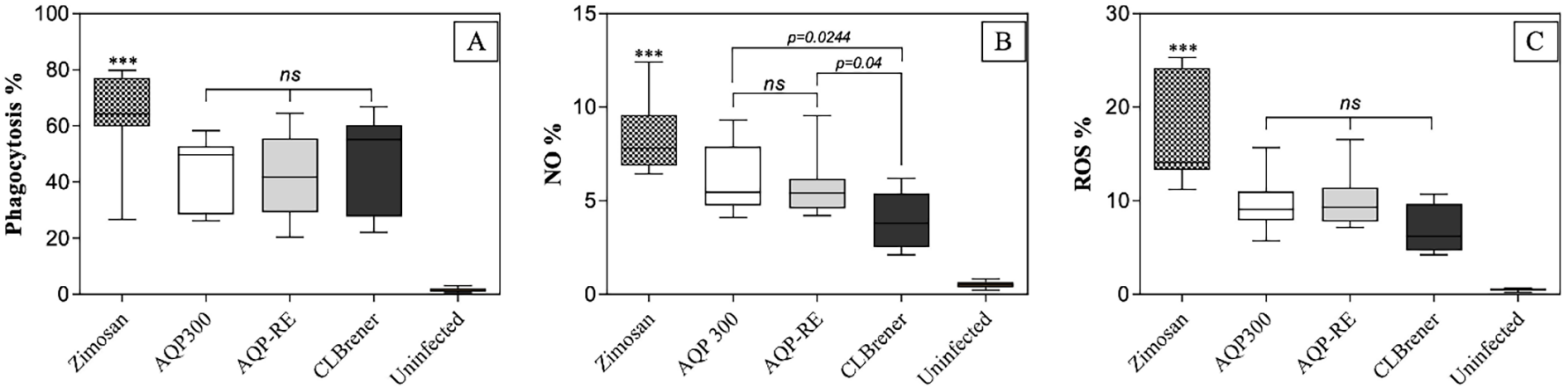
Phagocytosis **(A)**, nitric oxide (NO) production **(B)**, and reactive oxygen species (ROS) generation **(C)** by macrophages infected with *Trypanosoma cruzi* strains AQP-RE, AQP300, and the CL Brener clone. Within each panel, lines denote statistically significant differences (p < 0.05) or non-significant comparisons (ns) among the *T. cruzi* strains or clone groups analyzed. Data represent mean ± SEM from three independent experiments, analyzed using the non-parametric Mann–Whitney test. The symbol (***) indicates a statistically significant difference compared with the control group (Zymosan A). ns = non significant.

To establish a robust foundation for downstream analyses, we first conducted preliminary validation steps to confirm the experimental model and strain behavior. A reactivation protocol using *Triatoma infestans* successfully restored the infectivity of the originally non-virulent Arequipa strain (AQP300), yielding the reactivated strain (AQP-RE), which exhibited parasitemia levels comparable to the virulent CL Brener clone (**Supplementary Figure 1**). Genomic and Bioinformatic analysis confirmed the conservation of seven key antioxidant genes in the Arequipa strain genome, indicating that observed phenotypic differences in antioxidant responses likely stem from transcriptional regulation rather than gene loss (**Supplementary Figure 2**). Additionally, flow cytometric immunophenotyping of peritoneal exudate cells from C57BL/6 mice validated macrophages with the MHCII^+^Ly6C⁻ phenotype as the predominant cell population, supporting their selection for subsequent infection assays (**Supplementary Figure 3**). Functional assays using these validated macrophages revealed comparable phagocytosis rates for CL Brener, AQP-RE, and AQP300 strains (46.4 ± 16.1%, 42.5 ± 13.9%, and 44.0 ± 11.7%, respectively; **Figure 1A**). However, significant differences (p < 0.05) were observed in intracellular effector responses such as NO production. In this sense, macrophages infected with AQP-RE and AQP300 exhibited significantly higher nitric oxide (NO) production (5.7 ± 1.6% and 6.2 ± 1.8%, respectively) than those infected with CL Brener (3.9 ± 1.5%) (**Figure 1B**). On the other hand, the production of reactive oxygen species (ROS) did not have significant differences, but showed some increase in macrophages infected with AQP-RE (9.8 ± 2.9%) and AQP300 (9.5 ± 2.9%) compared to CL Brener (7.0 ± 2.5%) (**Figure 1C**). These results suggest that, although phagocytic uptake was similar, the lower production of NO and ROS in response to CL Brener infection could reflect a more effective antioxidant response by this virulent strain, facilitating intracellular survival.

### Gene expression of antioxidant enzymes

**Figure 2:**
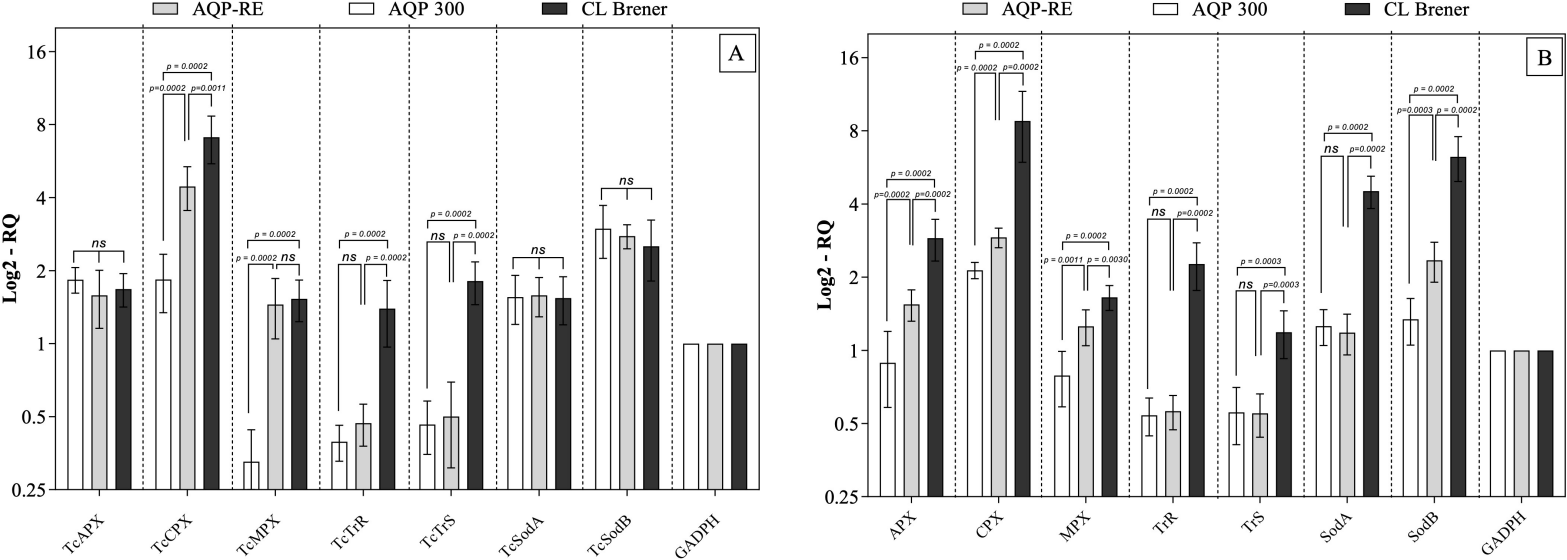
Relative expression of antioxidant enzymes in peritoneal macrophages infected with *Trypanosoma cruzi* strains AQP-RE, AQP300, and the CL Brener clone, quantified by RT-PCR. Panels show gene expression levels at **(A)** 3 hours post-infection (hpi) and **(B)** 24 hpi. Lines indicate statistically significant differences (p < 0.05) or non-significant comparisons (ns) between the evaluated *T. cruzi* strains or clone groups analyzed. Data represent mean ± SEM from three independent experiments, analyzed using the non-parametric Mann–Whitney test and expressed as fold change relative to the control group (trypomastigotes from culture) normalized to GAPDH.

To investigate the early antioxidant response of *T. cruzi* strains with differing virulence profiles, the expression of key antioxidant enzymes was quantified in macrophages infected with CL Brener, AQP-RE, or AQP300 at 3- and 24-hours post-infection. Macrophages infected with the virulent CL Brener clone exhibited significantly elevated transcript levels (p < 0.05) for multiple redox-regulatory genes, including TcAPX (ascorbate peroxidase), TcCPX (cytosolic tryparedoxin peroxidase), TcMPX (mitochondrial tryparedoxin peroxidase), TcTR (trypanothione reductase), TcTS (trypanothione synthetase), and both cytosolic and mitochondrial TcSODs (superoxide dismutases) (**Figures 2A–B**) (**Supplementary Table 3**). These enzymes form the core of the parasite’s antioxidant defense system and are critical for neutralizing host-derived reactive oxygen and nitrogen species. In contrast, the AQP-RE strain displayed a moderate but statistically significant upregulation of select antioxidant genes (TcAPX, TcCPX, TcMPX, and TcSOD-B) relative to the avirulent AQP300 strain (p < 0.05), suggesting partial activation of antioxidant mechanisms. The lowest expression levels were consistently observed in AQP300-infected macrophages, aligning with its inability to establish systemic infection *in vivo*. These results support the hypothesis that early overexpression of antioxidant enzymes facilitates parasite survival under oxidative stress and is a key feature of virulence in *T. cruzi*. Reduced antioxidant gene expression in the AQP300 strain likely contributes to its attenuated phenotype and limited replicative capacity within host cells.

### Parasitemia and parasite load in C57BL/6 mice

**Figure 3:**
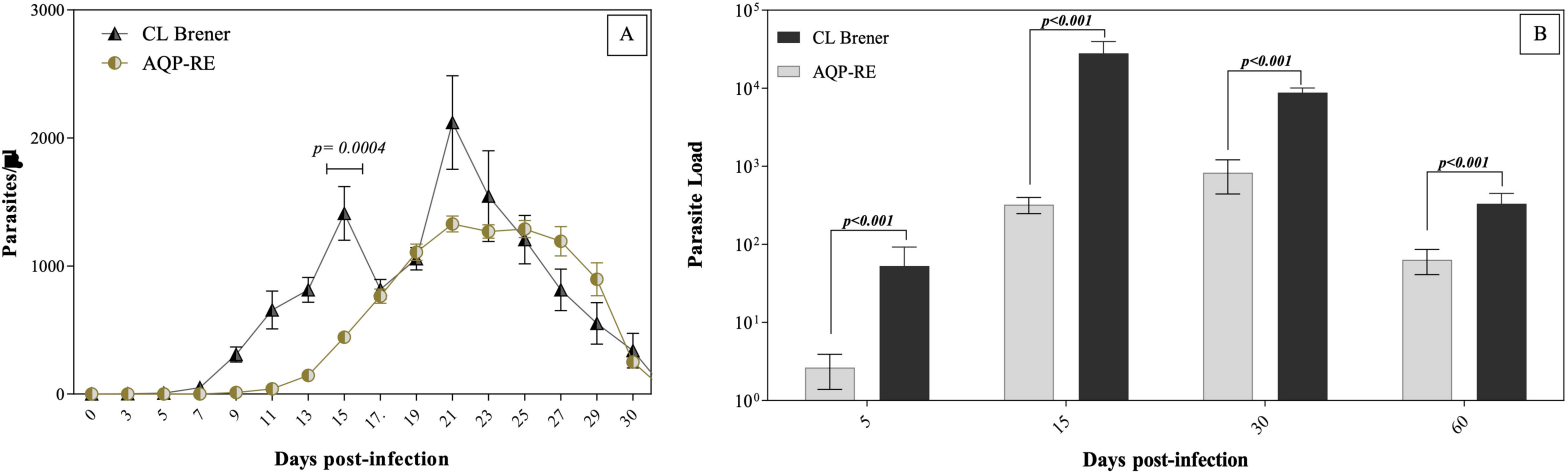
Infection dynamics in C57BL/6 mice inoculated intraperitoneally with the AQP-RE strain or the CL Brener clone of Trypanosoma cruzi. **(A)** Parasitemia levels were determined by counting motile trypomastigotes in 5 µL of tail blood at the indicated days post-infection. **(B)** Cardiac parasite burden expressed as parasite equivalents per milligram of tissue (Eq-P/mg), quantified by real-time PCR using a standard curve generated from serial dilutions of *T. cruzi* DNA. Within each panel, the indicated p-values represent statistically significant differences (p < 0.05) between groups. Data represent mean ± SEM from three independent experiments analyzed using the non-parametric Mann–Whitney test.

In this study, *in vivo* infection assays using C57BL/6 mice revealed that systemic infection was established exclusively by the CL Brener and AQP-RE strains. In contrast, the parental AQP300 strain failed to induce detectable bloodstream trypomastigotes. Parasitemia became detectable as early as 5 days post-infection (dpi) in both groups. The CL Brener clone displayed a biphasic parasitemia profile, with prominent peaks observed at 15 and 21 dpi (443.2 ± 105.8 and 1328.4 ± 187.4 parasites/μL, respectively). In contrast, AQP-RE exhibited a delayed onset of parasitemia and a single peak at 21 dpi (1328.4 ± 187.4 parasites/μL) and maintaining detectable parasitemia levels for approximately six days before declining. Notably, parasitemia at day 15 was significantly higher in mice infected with CL Brener (1410.4 ± 631.6 parasites/μL) compared to those infected with AQP-RE (443.2 ± 105.8 parasites/μL) (p < 0.05) (**Figure 3A**). During CL Brener infection, parasitemia peaked earlier and showed a more pronounced rise and fall in blood parasite count. Therefore, while overall parasitemia values for AQP-RE were lower compared to CL Brener, the peak was more prolonged and occurred later. In the same way, the quantitative PCR analysis of cardiac tissue revealed that the CL Brener clone exhibited markedly higher tissue tropism and parasite proliferation at all evaluated time points (p < 0.05). Parasite equivalents per milligram (Eq-P/mg) of cardiac tissue reached 52.8 ± 39.8 at 5 dpi, sharply increasing to 28,037.8 ± 11,659.5 at 15 dpi, then decreasing to 8,826.7 ± 1,275.6 at 30 dpi and 331.5 ± 117.9 at 60 dpi. In contrast, AQP-RE-infected mice showed significantly lower parasite loads: 2.7 ± 1.3, 322.8 ± 75.6, 829.3 ± 385.9, and 63.5 ± 22.5 Eq-P/mg at the same respective time points (**Figure 3B**). These results confirm the enhanced virulence and persistence of the CL Brener clone in host tissues and highlight the attenuated phenotype of the AQP-RE strain, despite its partial capacity to infect *in vivo*.

### Cytokine expression in cardiac tissue

**Figure 4:**
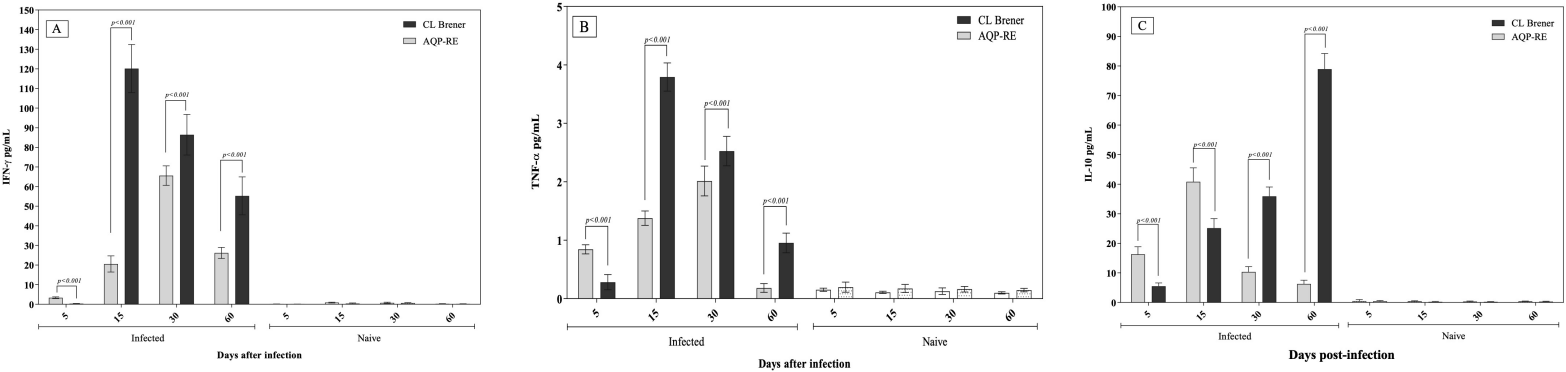
Cytokine expression levels in cardiac tissue of C57BL/6 mice infected with the AQP-RE strain or the CL Brener clone of *Trypanosoma cruzi*, quantified by RT-qPCR. Panels show relative expression of IFN-γ **(A)**, TNF-α **(B)**, and IL-10 **(C)** normalized to the murine β-actin gene and expressed as fold change relative to uninfected controls. Data represent mean ± SEM from three independent experiments analyzed using the non-parametric Mann–Whitney test. Within each panel, indicated p-values denote statistically significant differences (p < 0.001) between infected groups.

Temporal analysis of cytokine transcript levels in cardiac tissue revealed distinct immunological profiles associated with *T. cruzi* strain virulence. At 5 days post-infection (dpi), mice infected with the moderately virulent AQP-RE strain exhibited significantly higher expression of pro-inflammatory cytokines IFN-γ and TNF-α compared to those infected with the virulent CL Brener clone (p < 0.05) (**Figures 4A, B**). This early cytokine response likely reflects increased innate immune activation in response to the parasite’s reduced antioxidant defenses. However, this pattern was reversed at later time points (15, 30, and 60 dpi), when CL Brener-infected mice displayed significantly elevated IFN-γ and TNF-α expression (p < 0.05), consistent with ongoing parasite replication and chronic tissue inflammation. In contrast, expression of the anti-inflammatory cytokine IL-10 followed an inverse pattern. AQP-RE-infected mice showed higher IL-10 transcript levels at 5 and 15 dpi, suggesting early immunoregulatory activity that may contribute to limited tissue pathology. At 30 and 60 dpi, however, IL-10 expression was significantly higher in CL Brener-infected animals (p < 0.05) (**Figure 4C**) (**Supplementary Table 4**), indicative of a delayed compensatory immune regulation in response to persistent inflammation and parasitemia. These findings highlight a time-dependent shift in host cytokine responses shaped by parasite virulence. The ability of the CL Brener clone to sustain both pro-inflammatory and regulatory cytokine expression over time reflects its capacity to modulate the host immune environment, thereby enabling chronic infection and promoting cardiac pathology, a hallmark of Chagas disease progression.

## Discussion

This study provides mechanistic evidence that early antioxidant responses, measured both functionally and at the gene expression level, are closely associated with *T. cruzi* virulence. By comparing the highly virulent CL Brener clone with the strain isolated from Arequipa, Peru, both with the non-virulent (AQP300) and with the reactivated (AQP-RE) strains, we demonstrate that the specific differences in the handling of oxidative stress are related to the infective persistence of the parasite and the immune regulation in the host. The predominance of MHCII^+^Ly6C⁻ macrophages in the peritoneal exudate confirms the use of a mature, post-recruitment population with low baseline activation but high antigen-presenting capacity. This phenotype reflects cells in the resolution phase of inflammation, aligning with established peritoneal macrophage maturation patterns. Recognizing this profile is methodologically important, as baseline activation influences oxidative responses (**Supplementary Figure 3**). Within this context, macrophages infected with AQP300 and AQP-RE displayed significantly higher nitric oxide production despite similar phagocytosis rates. Although AQP-RE did not show a statistically significant increase in reactive oxygen species (ROS), this trend may indicate early modulation of oxidative stress preceding measurable antioxidant responses. This aligns with prior studies showing that non-virulent *T. cruzi* strains elicit more robust macrophage activation, resulting in effective restriction of parasite replication during early infection stages (Paiva and Bozza, 2014; Reverte et al., 2022; San Francisco et al., 2022). Conversely, infection with the CL Brener clone resulted in significantly lower intracellular NO and ROS levels, implying a more effective antioxidant response that enables the parasite to persist and proliferate within host cells. These findings are consistent with the observed upregulation of key antioxidant enzymes, such as TcAPX, TcCPX, TcMPX, TcTR, and Fe-SODs, in macrophages infected with CL Brener, reflecting an enhanced capacity to detoxify reactive intermediates. The ability of virulent *T. cruzi* strains to preemptively neutralize oxidative stress likely provides a critical window for intracellular establishment and dissemination and may contribute to chronic infection outcomes characterized by sustained tissue inflammation and parasite persistence.

The gene expression analysis of antioxidant enzymes provides strong support for the hypothesis that enhanced oxidative stress tolerance contributes to the virulence of *T. cruzi*. Notably, the virulent CL Brener clone exhibited significantly elevated expression of critical antioxidant genes, including tryparedoxin peroxidases (TcCPX, TcMPX), ascorbate peroxidase (TcAPX), and mitochondrial/cytosolic superoxide dismutases (TcSODs), particularly at 24 hours post-infection. These enzymes are integral to the parasite’s redox homeostasis system, neutralizing reactive oxygen and nitrogen species generated by host macrophages and facilitating intracellular survival (MaChado et al., 2012; Mesias et al., 2019; Paes et al., 2020; Wilkinson et al., 2008). The intermediate expression levels observed in AQP-RE, and the consistently low levels in the non-virulent AQP300 strain, reinforce a direct association between antioxidant capacity and parasite virulence. These findings corroborate prior studies that link heightened antioxidant gene expression to increased pathogenicity, immune evasion, and long-term persistence in host tissues (MaChado-Silva et al., 2016; Maldonado et al., 2021; Piacenza et al., 2008).

*In vivo* infection assays further substantiated these molecular trends. While both AQP-RE and CL Brener successfully established systemic infections in C57BL/6 mice, the original AQP300 isolate remained avirulent. CL Brener-infected mice exhibited higher peak parasitemia and significantly greater cardiac parasite burden across all time points compared to AQP-RE, consistent with the superior antioxidant defense profile that likely promotes early immune evasion and dissemination (Fernandes and Andrews, 2012; Rodriguez-Bejarano et al., 2021). The delayed and sustained peak of parasitemia observed in the AQP-RE strain suggests a different *in vivo* replication dynamic than that of CL Brener. Previous studies have reported that some *T. cruzi* isolates from endemic regions exhibit slower growth rates and prolonged parasitemia phases, associated with attenuated virulence and a more balanced host-parasite relationship (de Souza et al., 2010). In this context, the AQP-RE strain could establish infection by inducing a less aggressive systemic inflammatory response, allowing the parasite to maintain moderate parasitemia for a longer period without triggering rapid elimination by the host. These kinetic profiles are characteristic of strains that can persist longer in tissues, promoting chronic infection without causing extensive pathology in the acute phase. This is consistent with our additional findings on antioxidant responses and macrophage functional profiles, suggesting that parasite persistence may involve both reduced replication pressure and modulation of host immune activation.

Cytokine profiling in cardiac tissue reflected distinct temporal patterns associated with parasite virulence and host immune modulation. During the early phase of infection (5 dpi), AQP-RE induced a rapid increase in IFN-γ and TNF-α expression, consistent with strong innate immune activation and limited capacity to suppress early inflammation. In contrast, the CL Brener clone elicited a delayed but sustained upregulation of these pro-inflammatory cytokines at later stages, accompanied by increased IL-10 expression, suggesting a more controlled and modulated inflammatory response that favors parasite persistence and chronic infection. Our results are consistent with previous reports describing the temporal modulation of cytokine responses during *T. cruzi* infection. highlighted that early production of IFN-γ and TNF-α reflects a strong innate response associated with parasite control, whereas delayed or sustained expression accompanied by IL-10 supports immune regulation and chronic persistence. Similarly, Macaluso et al. (2023) emphasized that parasite antioxidant defenses, such as tryparedoxin peroxidases and Fe-SODs, enable evasion of oxidative stress and influence the timing of host cytokine activation. In agreement with these studies, the AQP-RE strain in our model elicited an early inflammatory burst, while the virulent CL Brener clone induced a delayed but sustained cytokine response, linking enhanced antioxidant capacity to immune modulation and increased persistence (Acevedo et al., 2018; Cardillo et al., 2015; Macaluso et al., 2023). This dysregulated cytokine milieu is characteristic of chronic Chagas cardiomyopathy and may result from the parasite’s manipulation of oxidative and immune pathways to maintain tissue tropism and persistence.

Our results may align with the contrasting macrophage proteomic findings of Herreros-Cabello et al. (2024) (Herreros-Cabello et al., 2024), who observed enhanced TLR7/9 signaling with the lethal Y strain and IL-11 activation with the non-lethal VFRA strain, noting that VFRA possesses more antioxidant molecules than the Y strain. While this may counter our observation that high antioxidant gene expression correlates with virulence, these datasets likely capture different regulatory layers. Our data indicate a transient, inducible antioxidant burst in virulent parasites early post-infection, whereas VFRA’s constitutive antioxidant load reflects a persistence-oriented adaptation. Together, these studies suggest that inducibility, not abundance per se, links antioxidant capacity to virulence, with strain/DTU context and host background further modulating the outcome. However, to corroborate and expand upon these findings, future studies should include a broader panel of *T. cruzi* strains and integrate complementary proteomic and functional inhibition analyses to validate whether the observed transcriptional patterns correspond to protein-level regulation and enzymatic activity. Although our findings reveal a potential mechanistic link between antioxidant activity and immune evasion, the present study is limited by the number of strains examined and the lack of proteomic validation.

Taken together, our findings suggest that early oxidative activity, both host-derived and parasite-driven, may serve as an indicator of *T. cruzi* virulence. The combined assessment of macrophage-derived NO/ROS production and parasite antioxidant gene expression during the initial phase of infection could contribute to strain characterization and risk evaluation in Chagas disease. While our results support a link between antioxidant mechanisms and infection dynamics, we recognize that additional factors, such as metabolic adaptation, immune evasion strategies, or tissue-specific interactions, may also influence the observed phenotypes. Therefore, the parasite’s antioxidant machinery should be considered a potential, though not exclusive, determinant of virulence and a promising avenue for future therapeutic investigation.

## Data Availability

The original contributions presented in the study are included in the article/**Supplementary Material**. Further inquiries can be directed to the corresponding author.
