# Supplementary material for: Differential antioxidant enzyme profiles reveal early molecular signatures of virulence in Trypanosoma cruzi DTU-TcI and DTU-TcVI strains
Source: Front Cell Infect Microbiol. 2025 Nov 28;15:1664827. doi: 10.3389/fcimb.2025.1664827 (PMC12698546; doi:10.3389/fcimb.2025.1664827)
Supplement: Supplementary file 1 [file DataSheet1.docx]

**Differential Antioxidant Enzyme Profiles Reveal Early Molecular Signatures of Virulence in Trypanosoma cruzi DTU-TcI and DTU-TcVI Strains**

**Edward Valencia Ayala^1,3^, João Reis Cunha^4^, Maritza Calderón Sánchez^2^, Angela Giovanna Vidal Riva^3^, Daniella Castanheira Bartholomeu^4^, Alexandre Ferreira Marques^5^**

**^1^**Laboratorio de Parasitología Molecular y Celular, Facultad de Ciencias Biológicas, Universidad Nacional Mayor de San Marcos, Lima-Perú

**^2^**Laboratorio de Investigación en Enfermedades Infecciosas - Laboratorio de Biología Molecular, Facultad de Ciencias y Filosofía, Universidad Peruana Cayetano Heredia, Lima-Perú.

**^3^**Instituto de Investigación - Centro de Investigación en Virología, Facultad de Medicina Humana, Universidad de San Martin de Porres, Lima-Perú.

**^4^**Laboratório de Imunologia e Genômica de Parásitos - Departamento de Parasitologia, Instituto de Ciências Biológicas/ICB, Universidade Federal de Minas Gerais, Minas Gerais-Brazil.

**^5^**Center for Molecular and Cellular Biosciences, School of Biological, Environmental, and Earth Sciences, University of Southern Mississippi, Hattiesburg, MS, USA

# Supplementary Figure 1. Arequipa strain infectivity reactivation: Triatoma infestans nymphs (stage 5) were artificially fed with blood containing Epimastigotes (10⁶ parasites/mL). After development to the adult stage, metacyclic trypomastigotes were recovered from their intestinal content and used to infect mice. Once parasitemia was confirmed in infected mice, the reactivated parasites were used to antioxidant studies.


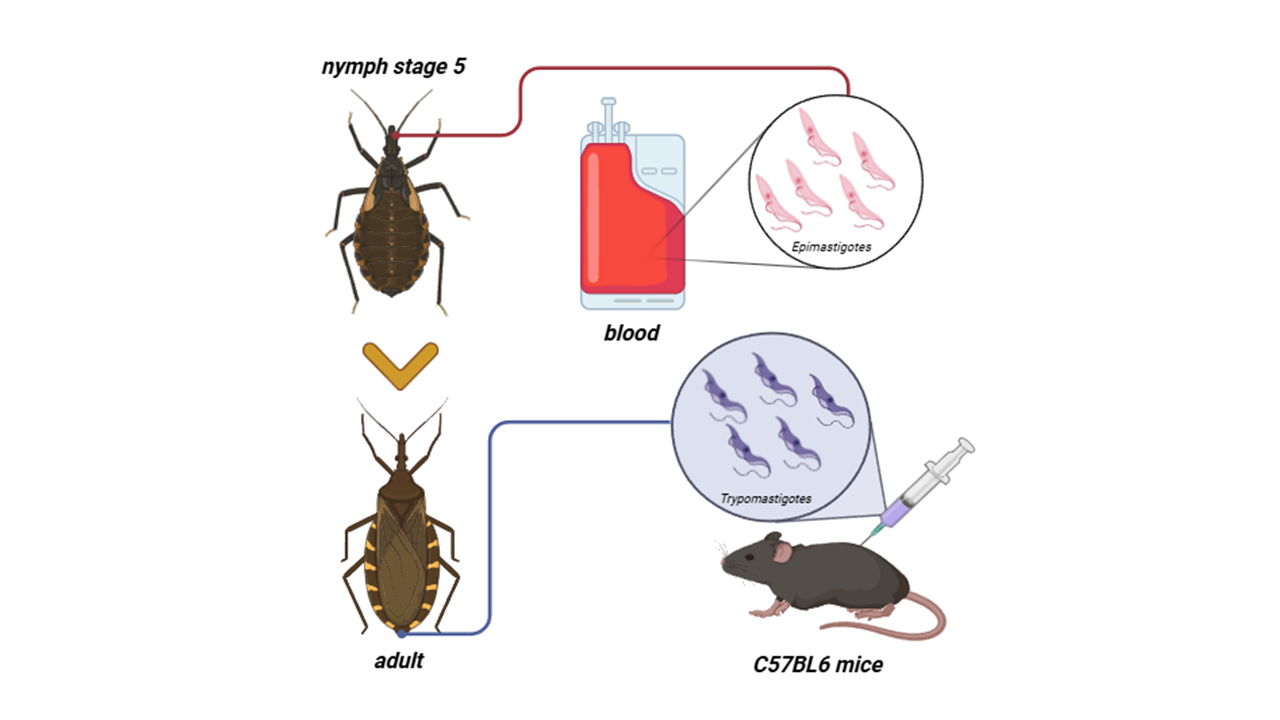


**Supplementary Figure 2. Pipeline about mapping reads, assembly and design of primers for antioxidants genes:** Genomic and bioinformatic analysis of the parasite genome confirmed the conservation of the sequences of five key antioxidant genes, from which primers were designed to amplify these genes.


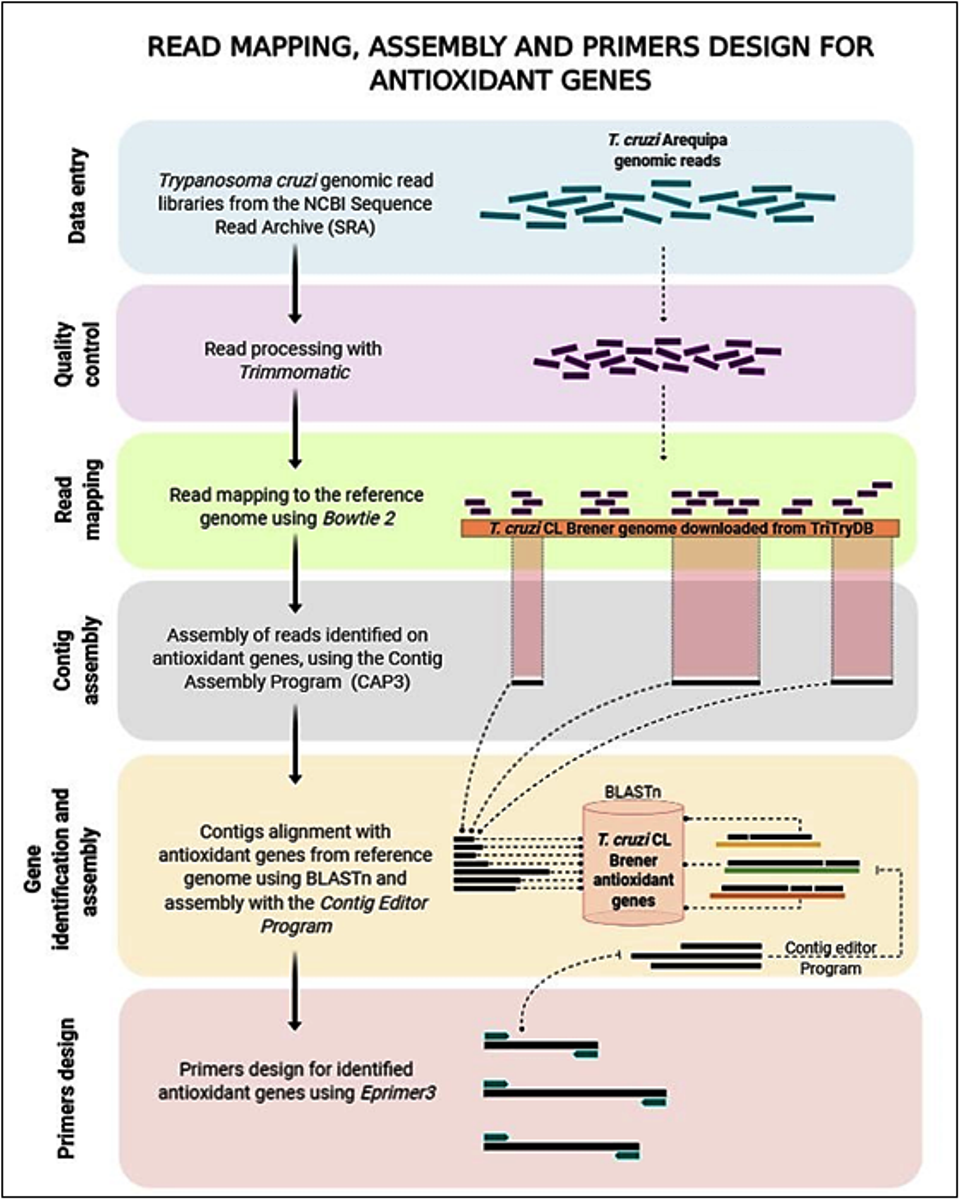


**Supplementary Figure 3.** **Immunophenotyping into peritoneum cells from C57BL/6 mice: A)** Leukocytes selection through size versus granularity (SSC-A vs FSC-A), **B)** Singletes selection from Leukocytes population (FSC-H vs FSC-A), **C)** Specific Lymphocytes B (CD19+) population from Singletes (CD45+). **D)** Non-specific MHC-II+ cells from Singletes (CD45+), **E)** Specific Macrophages and Neutrophils (Ly6G-/Ly6G+) populations from MHC-II+ cells, **F)** Differential M1 and M2 Macrophages (Ly6C+/ Ly6C-) populations.


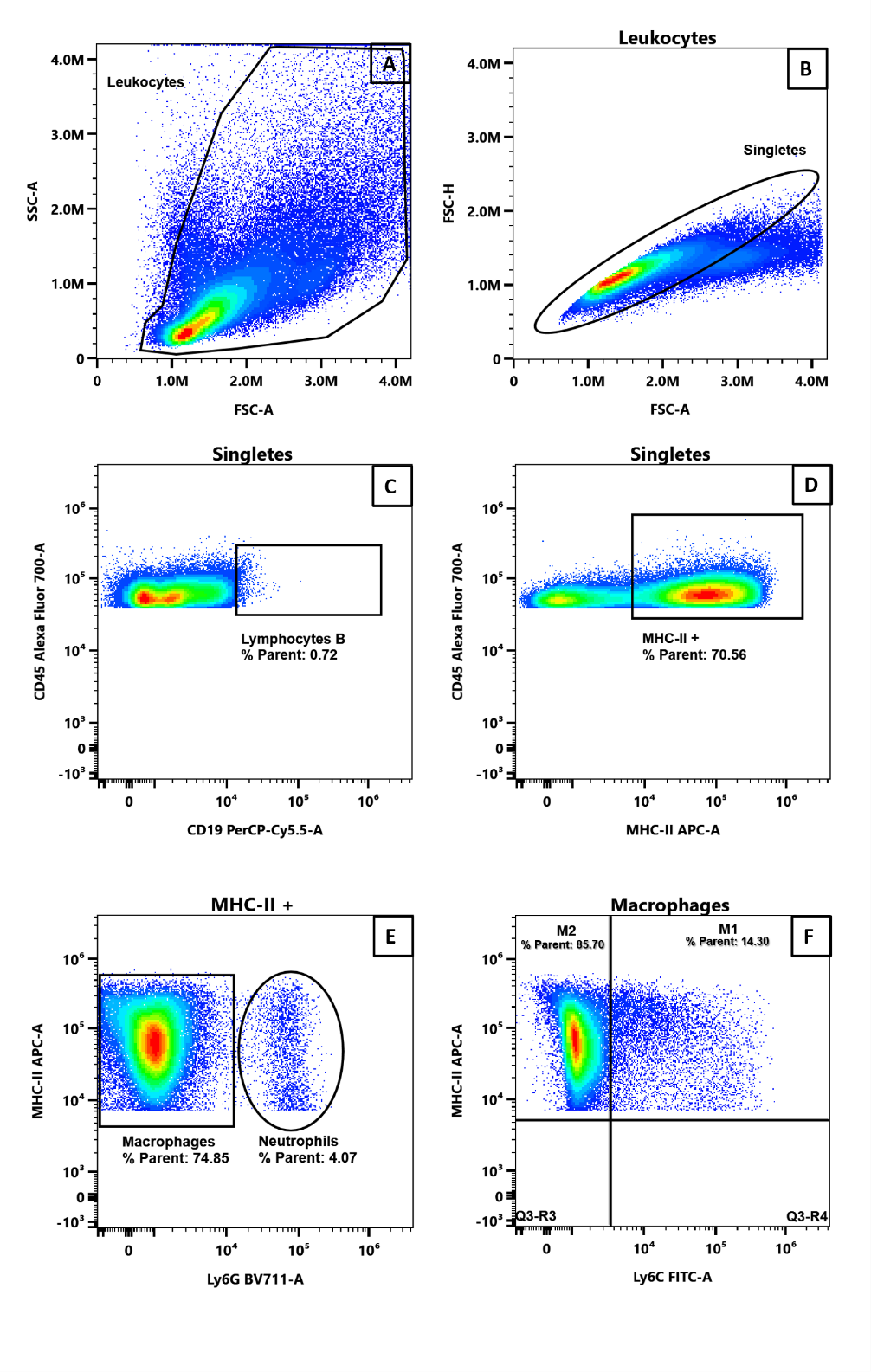


**Supplementary Table 1.**

**Table 1: Panel of fluorochrome-conjugated monoclonal antibodies used for flow cytometric immunophenotyping of murine peritoneal cells.**

This table lists the antibodies employed to identify specific immune cell populations. Each antibody is displayed with its corresponding fluorochrome label, clone designation, and the target cellular antigen or functional phenotype.

| **Antibody** | **Fluorochrome** | **Clone** | **Target phenotype** |
| --- | --- | --- | --- |
| **MHC-II** | APC - (Allophycocyanin) | M5/114.15.2 | Macrophage-Activation |
| **CD19** | PerCP/Cy 5.5 - (Peridinin Chlorophyll/Cyanine 5.5) | HIB19 | Lymphocyte B |
| **LY6G** | BV 711 - (Brillant Violet) | 1A8 | Neutrophil |
| **LY6C** | FITC - (Fluorescein Isothiocyanate) | HK1.4 | Macrophage polarization |
| **CD45** | AF 700 - (Alexa Fluor 700) | 30-F11 | Leukocytes |

**Supplementary Table 2.**

**Table 2: Primers for molecular expression of *T. cruzi* Antioxidant Enzymes.**

This table details the forward and reverse primer sequences used to amplify transcripts of five antioxidant enzymes in *T. cruzi*, along with the expected product sizes (in base pairs). The targets include ascorbate peroxidase (TcAPX), cytosolic and mitochondrial tryparedoxin peroxidases (TcCPX, TcMPX), trypanothione reductase (TcTR), trypanothione synthetase (TcTS) and iron-dependent superoxide dismutases (TcSodA, TcSodB).

| **ID** | **forward primer** | **reverse primer** | **size (pb)** |
| --- | --- | --- | --- |
| **TcAPX** | GGAAAAAGGATGGGAGTCCT | AAGGACCATAAATCGGCGTA | 151 |
| **TcCPX** | TCTACCCGATGGACTTCACC | CTCCTCCTTCAGCACACCAT | 239 |
| **TcMPX** | AGCCTTTTCAGATGCTCAGG | CGCAGAATCCCTTTATCGTC | 249 |
| **TcSodA** | GTGGACCCGTCGAATAAGAA | TGACCGACCAGATCTCCTTC | 169 |
| **TcSodB** | TGCGAAGTTCAAGGAGGAGT | AAGTTGGGCTCTGTCAGTGG | 144 |
| **TcTrR** | ATTGCATCAGCAGCAATGAG | TTGTGAGTTCCTCACGGAGA | 198 |
| **TcTrS** | CAAAACCATCGGTTTCACAA | CCGTTTGAAGTGAAGCGACT | 198 |

All primers were 5’ - 3’ orientation

**Supplementary Table 3.**

**Table 3: Expression of Antioxidant Enzymes.**

This table shows data on the expression of the antioxidant enzyme from the AQP300 and AQP-RE strains, as well as from the CL Brener clone of Trypanosoma cruzi. These values ​​were obtained 3 and 24 hours after initial infection. Furthermore, this table shows a p-value < 0.05, which represents a significant difference according to the Kruskal-Wallis test.

| **Antioxidant**  **Enzymes** | **Times after initial infection** | | | | | | | |
| --- | --- | --- | --- | --- | --- | --- | --- | --- |
|  | ***3 hpi*** | | | | ***24 hpi*** | | | |
|  | ***AQP300*** | ***AQP-RE*** | ***CL Brener*** | ***p value*** | ***AQP300*** | ***AQP-RE*** | ***CL Brener*** | ***p value*** |
|  |  |  |  |  |  |  |  |  |
| **TcAPX** | 1.84±0.22 | 1.58±0.4 | 1.68±0.3 | **0.2573** | 0.89±0.31 | 1.55±0.23*^a^* | 2.89±0.56*^a, a´^* | **< 0.0001** |
|  |  |  |  |  |  |  |  |  |
| **TcCPX** | 1.83±0.49 | 4.45±0.91*^a^* | 7.09±1.57 *^a, a´^* | **< 0.0001** | 2.14±0.16 | 2.91±0.27*^a^* | 8.78±2.84*^a, a´^* | **< 0.0001** |
|  |  |  |  |  |  |  |  |  |
| **TcMPX** | 0.33±0.12 | 1.45±0.40*^a^* | 1.53±0.29*^a^* | **0.0004** | 0.79±0.20 | 1.26±0.21*^a^* | 1.65±0.19*^a, a´^* | **0.0001** |
|  |  |  |  |  |  |  |  |  |
| **TcTrR** | 0.39±0.07 | 0.47±0.09 | 1.39±0.43*^a, a´^* | **0.0002** | 0.54±0.09 | 0.56±0.09 | 2.26±0.50*^a, a´^* | **0.0004** |
|  |  |  |  |  |  |  |  |  |
| **TcTrS** | 0.46±0.12 | 0.50±0.19 | 1.81±0.36 *^a, a´^* | **0.0005** | 0.56±0.15 | 0.55±0.11 | 1.19±0.26*^a, a´^* | **0.0007** |
|  |  |  |  |  |  |  |  |  |
| **TcSodA** | 1.56±0.36 | 1.58±0.29 | 1.54±0.35 | **0.9680** | 1.26±0.21 | 1.18±0.23 | 4.52±0.69*^a, a´^* | **0.0004** |
|  |  |  |  |  |  |  |  |  |
| **TcSodB** | 2.98±0.73 | 2.77±0.32 | 2.52±0.71 | **0.5234** | 1.34±0.29 | 2.35±0.44*^a^* | 6.26±1.31*^a, a´^* | **< 0.0001** |
|  |  |  |  |  |  |  |  |  |

The symbol represent significant difference: (*a*) in relation to AQP300 and (*a´*) in relation to AQP-RE.

**Supplementary Table 4.**

**Table 4: Values of cytokines expression by AQP-RE strain and CL Brener clone of T. cruzi.**

This table shows data on the Cytokine expression that were induced in heart during the infection of mice model C57BL6 with the AQP-RE strain and the CL Brener clone of Trypanosoma cruzi. These values ​​were obtained 5, 15, 30 and 60 days after initial infection. This table shows a p-value < 0.05, which represents a significant difference according to the Kruskal-Wallis test.

| **Infection Day** | **INFγ** | | **TNFα** | | **IL-10** | | ***P value*** |
| --- | --- | --- | --- | --- | --- | --- | --- |
|  | ***CL Brener*** | ***AQP-RE*** | ***CL Brener*** | ***AQP-RE*** | ***CL Brener*** | ***AQP-RE*** |  |
|  |  |  |  |  |  |  |  |
| 5 | 0.33 ± 0.09 | 3.35 ± 0.44*^cl^* | 0.28 ± 0.13 | 0.84 ± 0.08 *^cl^* | 5.51 ± 1.08 | 16.30 ± 2.54*^cl^* | **< 0.001** |
| 15 | 120.16 ± 12.25*ª^´^* | 20.55 ± 4.11 | 3.79 ± 0.24*ª^´^* | 1.38 ± 0.12 | 25.16 ± 3.20 | 40.84 ± 4.70*^cl^* | **< 0.001** |
| 30 | 86.44 ± 10.34*ª^´^* | 65.60 ± 4.97 | 2.52 ± 0.25*ª^´^* | 2.01 ± 0.26 | 35.91 ± 3.16*ª^´^* | 10.35 ± 1.78 | **< 0.001** |
| 60 | 55.27 ± 9.68*ª^´^* | 26.19 ± 2.81 | 0.95 ± 0.17*ª^´^* | 0.18 ± 0.07 | 78.94 ± 5.28*ª^´^* | 6.26 ± 1.27 | **< 0.001** |

The symbol represent significant difference: (*a´*) in relation to AQP-RE and (*cl*) in relation to CL Brener.

**Supplementary Information 1: Scaffold and reference genes of antioxidant enzymes**

**1. Ascorbate-dependent peroxidase (TcAPX)**

**Reference Gene: TcCLB.503745.30**

**>Scaffold AQP**

GCACCCCCGTTTGATGTGAGCTCACTAAGAAAGGACATTGAAGAAATACTCTCCGAGGACATGTCTAAAGGGCCTCTTTTTGTGCGACTCGCGTGGCACGAGGCTGGATCATGGGACTGCCGGAAAAAGGATGGGAGTCCTAATTCCGCTTCGATGCGATTTCACCCAGAGTGCAGCTATGCCGGCAACAAAGGGCTTGATAAAGGACGAACTGCGCTGGAATCGCTCAAGAAAAAGTACCCGAAAATTTCATACGCCGATTTATGGTCCTTCGCAGCGGTGGTGTCGATTGAGGCGATGGGCGGCCCGGAGATTCCGTGGCGGTGGGGGCGCGTGGATGCAAAGGACGGCAGCGTTTGTGGCCCCGATGGGCGTCTCCCTGACGCGTCCAGGATGCAGGATCACGTGCGGGATGTCTTTTCACGTCTTGGCTTTAATGATGAAGAAACTGTTGCCTTGATCGGGGCCCACACATGTGGTGAGTGCCATCTGGAGAACACGGGCTACGTGGGTCCGTGGACGCACGACAAGTACGGCTTTGACAACTCCTTTTTTACGGAGCTTTTTGGCAACGAGTGGATGTTGAACCCCAATGTGAATAAAATGCAGTTCATGGACAAAACCACTAACCGCCTTATGATGCTGCCCGCTGATGTTTCCATTCTTCTGGACGACAAATATCGTTCCATCGCGAAGAAGTACGCGGACGACAATGATTATTTTTGCAACGCCTTCAGCAAGGCTTATCAGAAGTTGCTTGAGGTTGGAACCACCAATCTCAAGTCTCTCCCAGCAGAGTCAAAATAGGCAAA

**>TcCLB.503745.30**

ATGGCTTTTTGTTTTGGTTCATTCTTTTCGAAGTACGCTTCATCCAAATCAGGTTCTCAGGCAAGGTACCGTTTTCTTCATTCCTCGGCTAAAATTGCTGCCGGCGCCACCGGTGCATTGCTTTTGGGCGGTGCCACCGTGGCCTTATGTTATTTTCCCTCGGGACGAAAGGTCACGGAGGCACCGCCGTTTGATGTGAACTCACTAAGAAGGGACATTGAAGAAATACTCTCCGAGGATATGTCTAAAGGGCCTCTTTTTGTGCGACTCGCGTGGCACGAGGCTGGATCATGGGACTGCCGGAAAAAGGATGGGAGTCCTAATTCCGCTTCGATGCGATTTCACCCAGAGTGCAGCTATGCCGGCAACAAAGGGCTTGATAAAGGACGAAATGCGCTGGAATCGCTCAAGAAAAAGTACCCGAAAATTTCATACGCCGATTTATGGTCCTTCGCAGCGGTGGTGTCGATTGAGGCGATGGGTGGCCCGGAGATTCCGTGGCGGTGGGGGCGCGTGGATGCAAAGGACGGCAGCGTTTGTGGCCCCGATGGGCGTCTCCCTGACGCGTCCAGGATGCAGGATCACGTGCGGGATGTCTTTTCACGTCTTGGCTTTAATGATGAAGAAACTGTTGCCTTGATCGGGGCCCACACATGTGGTGAGTGCCATCTGGAGAACACGGGCTACGTGGGTCCGTGGACGCACGACAAGTATGGCTTTGACAACTCCTTTTTTACGGAGCTTTTTGGCAACGAGTGGATGCTGAACCCCAATGTGAAGAAAATGCAGTTCATGGACAAAACCACTAACCGCCTTATGATGCTGCCCGCTGATGTTTCCATTCTTCTGGACGACAAGTATCGTTCCATCGCGAAGAAGTACGCGGACGACAATGATTATTTTTGCAACGCCTTCAGCAAGGCTTATCAGAAGTTGCTTGAGGTTGGAACCACCGATCTCAAGTCTCTCCCAGCAGAGTCAAAATAG

**2. Superoxide dismutase A (Tc_SodA)**

**Reference Gene: TcCLB.509775.40** 

**>Scaffold AQP**

CAGAGGGCGCATGGCGTTGATGAGCTACGCGACGCTGCCTGACCTTTTGAAACCAAGTGGTGCCCCGGCCGAGTTGCCCAAGTTAGGGTTTAACTGGAAGGATGGATGTGCCCCTGTCTTCAGCCCGCGCCAGATGGAATTGCACTACACCAAACACCACAAGGCGTATGTGGACAAATTAAATGCGCTGGCAGGGACAAAATACGATGGGAAGAGCATTGAGGAGATTATCCTTGCGGTCGCAAATGACGCTGAGAAGAAGGGTCTTTTCAACCAAGCCGCACAGCATTTTAACCACACCTTTTACTTTCGTTGTATCACCCCTAATGGGAAGGCGATGCCAAAGTCCCTTGAGTCTGCTGTTACGGCCCAGTTTGGTTCGGTGGAACAGTTTAAAGATGCCTTTGTGCAGGCAGGGGTGAATAACTTTGGCTCAGGTTGGACATGGCTGTGCGTGGACCCGTCGAATAAGAATCAGCTCGTTATTGACAATACGAGCAACGCCGGATGTCCCTTAACGAAGGGACTGCGCCCCGTCCTCGCGGTGGATGTGTGGGAGCATGCGTACTACAAGGACTTTGAAAACCGCCGGCCGGATTACTTGAAGGAGATCTGGTCGGTCATCGACTGGGAGTTTGTTGCAAAGATGCATGTGCAGGCAATAAAATAAAATA

**> TcCLB.509775.40**

ATGTTGAGACGTGCGGTGAATATATCCATTGCCAGAGGGCGCATGGCGTTGATGAGCTACGCGACGCTGCCTGACCTTTTAAAACCAAGTGGCGCCCCGGCCGAGTTGCCCAAGTTGGGGTTTAACTGGAAGGATGGATGTGCCCCTGTCTTCAGCCCGCGCCAGATGGAATTGCACTACACCAAACACCACAAGGCGTATGTGGACAAATTAAATGCGCTGGCAGGGACAACATACGATGGGAAGAGCATTGAGGAGATTATCCTTGCGGTCGCAAATGACGCCGAGAAGAAGGGTCTTTTCAACCAAGCCGCACAGCATTTTAACCACACCTTTTACTTCCGTTGCATCACCCCTAATGGGAAGGCGATGCCAAAGTCCCTTGAGTCTGCTGTTACGGCCCAGTTTGGTTCGGTGGAACAGTTTAAAGATGCCTTTGTGCAGGCAGGGGTGAATAACTTTGGCTCGGGTTGGACGTGGCTGTGCGTGGACCCGTCGAATAAGAATCAGCTCGTTATTGACAATACGAGCAACGCCGGATGTCCCTTAACGAAGGGACTGCGCCCCGTCCTCGCAGTGGATGTGTGGGAGCATGCGTACTACAAGGACTTTGAAAACCGCCGGCCGGATTACTTGAAGGAGATCTGGTCGGTCATCGACTGGGAGTTTGTTGCAAAGATGCATGCGCAGGCAATAAAATAA

**3. Superoxide dismutase B (TcSodB)**

**Reference Gene: TcCLB.511715.10**

**>Scaffold AQP**

ATGGTCTTCAGCATTCCTCCGCTCCCATGGGGCTACGATGGGCTTGCGGCAAAAGGCCTCTCAAAGCAGCAGGTGACGCTCCACTACGACAAACACCATCAGGGGTATGTGACGAAACTCAACGCCGCGGCGCAGACAAACTCCGCGCTTGCAACGAAAAGCATCGAGGAAATCATCAGGACGGAGAAAGGCCCCATCTTCAACCTTGCGGCGCAGATTTTTAACCACACGTTCTACTGGGAGAGCATGTGTCCTAATGGCGGTGGCGAGCCGACGGGAAAAGTTGCCGACGAGATCAACGCTTCATTTGGCAGTTTTGCGAAGTTCAAGGAGGAGTTTACAAACGTGGCTGTGGGCCACTTTGGCTCGGGTTGGGCGTGGCTTGTGAAGGACACCAATTCCGGCAAACTGAAAGTCTACCAGACGCATGACGCGGGATGTCCACTGACAGAGCCCAACTTGAAGCCTCTCCTTACATGCGATGTATGGGAGCATGCGTACTACGTGGACTACAAGAACGACCGTGCGGCATATGTGCAGACCTTTTGGAACGTTGTCAACTGGAAGGACGTGGAACGCGTCTCAGTGAGACTAGGTACGTGCATTTGCGGAGTTATTTGTAGTTGCATCTCCACAACCATACCTCCAATTTTTCTCTCTTCACGCACGAATTGCATCATGTGTTGTAAGGGCTTTGGT

**>TcCLB.511715.10**

ATGGTCTTCAGCATTCCTCCGCTCCCATGGGGCTACGATGGGCTTGCGGCAAAAGGCCTCTCAAAGCAGCAGGTGACGCTCCACTACGACAAGCACCATCAGGGGTATGTGACGAAACTCAACGCTGCGGCGCAGACAAACTCCGCGCTTGCAACGAAGAGCATCGAGGAGATCATCAGGACGGAGAAAGGCCCCATCTTCAACCTTGCGGCGCAGATTTTTAACCACACGTTCTACTGGGAGAGCATGTGTCCTAATGGCGGTGGCGAGCCGACGGGAAAACTTGCCGACGAGATCAACGCTTCATTTGGCAGTTTTGCGAAGTTCAAGGAGGAGTTTACAAACGTGGCTGTGGGCCACTTTGGCTCGGGTTGGGCGTGGCTTGTGAAGGACACCAATTCCGGCAAACTGAAGGTCTACCAGACGCATGACGCGGGATGTCCACTGACAGAGCCCAACTTGAAGCCTCTCCTTACATGCGATGTATGGGAGCATGCGTACTACGTGGACTACAAGAACGACCGTGCGGCATACGTGCAGACCTTTTGGAACGTTGTCAACTGGAAGAACGTGGAACGGCGTCTCAGTGAGACTGGGTACGTGCATTTGCGGAGTTATTTGTAG

**4. Trypanothione reductase (TcTrR)**

**Reference Gene: TcCLB.504507.5**

**>Scaffold AQP**

ATTGCTTCCCCCTTACAATATTCTACAAGAATCATGATGCCAAAAATTTTTGATTTGGTTGTCATTGGCGCCGGCTCGGGCGGACTGGAGGCTGCTTGGAACGCGGCGACACTCTACAAGAAGCGGGTTGCGGTGATTGATGTTCAGATGGTTCACGGGCCCCCGCGTTTTTTTTCTGCTCTAGGCGGCACGTGTGTCAATGTTGGCTGCGTTCCGAAGAAATTGATGGTTACAGGGGCCCAATACATGGAGCACCTGCGCGAGTCTGCTGGGTTCGGGTGGGAGTTTGATCGCACCACTCTCAGGGCGGAATGGAAGAAACTTATTGCTGTCAAGGACGAGGCGGTGCTGAATATCAACAAGAGTTATGACGAGATGTTTCGGGACACGGAGGGTCTGGAGTTTTTCCTGGGCTGGGGATCACTGGAGTCAAAGAACTGTCGGTCAATGTTCGCGAGAGTGCCGACCCGGCCAGCGCAGTTGAAGGAGCGCCTGGAGACGGAGCACATTCTACTTGCCAGTGGGTCGTGGCCGCACATGCAAAACATCCCTGGTATTGAGCATTGCATCAGCAGCAATGAGGCATTCTACCTGCCGGAGCCACCGCGTCGTGTCCTCACTGTCGGCGGAGGCTTCATTTCCGTGGAGTTTGCCGGCATTTTTAACGCCTACAAGCCGAAGGACGGACAAGTGACGTTGTGCTACCGCGGTGAAATGATCCTTCGTGGCTTTGACCACACTCTCCGTGAGGAACTCACAAAGCAGCTCACCGCCAACGGCATTCAAATCCTTACGAAGGAAAATCCGGCCAAGGTGGAGTTGAACGCGGATGGCAGCAAAAGTGTTACTTTCGAGAGCGGCAAAAAGATGGACTTTGATCTTGTCATGATGGCGATTGGCCGTTCTCCCCGAACCAAGGATTTACAGCTGCAAAACGCCGGCGTCATGATCAAAAACGGTGGTGTGCAGGTGGACGAGTACTCGCGCACGAATGTTTCCAACATTTACGCCATCGGTGACGTCACAAATCGTGTCATGTTGACACCCGTGGCCATAAATGAAGCCGCTGCCCTTGTGGATACAGTCTTTGGTACCACTCCGCGAAAGACGGACCACACCCGTGTGGCGAGTGCCGTCTTCTCTATTCCTCCAATTGGTACCTGCGGTCTCATTGAAGAGGTTGCATCCAAGCGCTACGAGGTGGTGGCGGTATACCTTTCCAGCTTTACCCCGCTCATGCACAAAGTCAGCGGATCAAAGTATAAGACTTTTGTTGCAAAGATAATTACCAACCACTCCGATGGCACTGTTCTTGGTGTACATCTTCTTGGGGACAATGCCCCAGAAATCATCCAAGGTATTGGTATCTGTCTCAAGTTAAACGCCAAAATATCCGACTTCTACAACACTATTGGTGTGCATCCCACAAGTGCGGAGGAGCTGTGCTCCATGCGCACTCCTTCTTACTACTATGTTAAAGGTGAGAAGATGGAAAAGCCTTCAGAGGCATCTCTGTAAGGGAAGAGG

**>TcCLB.504507.5**

ATGTCAAAAATTTTTGATTTGGTTGTCATTGGCGCCGGCTCGGGCGGACTGGAGGCTGCTTGGAACGCGGCGACACTCTACAAAAAGCGGGTTGCGGTGATTGATGTTCAGATGGTTCACGGGCCCCCGTTTTTTTCTGCTCTAGGCGGCACGTGTGTCAATGTTGGCTGCGTTCCGAAGAAATTGATGGTTACAGGGGCCCAATACATGGAACACCTACGCGAGTCTGCTGGGTTCGGGTGGGAGTTTGATCGCACCACTCTCAGAGCGGAATGGAAGAAACTTATTGCTGTCAAGGACGAGGCGGTGCTGAATATCAACAAGAGTTATGAGGAGATGTTTCGGGACACGGAGGGTCTGGAGTTTTTCCTGGGCTGGGGATCACTGGAGTCAAAGAATGTCGTCAATGTTCGCGAGAGTGCCGACCCGGCCAGCGCAGTGAAGGAGCGCCTGGAGACGGAGCACATTCTACTTGCCAGTGGGTCGTGGCCGCACATGCCAAACATCCCTGGTATTGAGCATTGCATCAGCAGCAATGAGGCATTCTACCTGCCGGAGCCACCGCGTCGTGTCCTCACTGTCGGCGGAGGCTTCATTTCCGTGGAGTTTGCAGGCATTTTTAACGCCTACAAGCCGAAGGACGGACAAGTGACGTTGTGCTACCGCGGTGAAATGATCCTTCGTGGCTTTGACCACACTCTCCGTGAGGAACTCACAAAGCAGCTCACCGCCAACGGCATTCAAATCCTTACGAAGGAAAATCCGGCCAAGGTGGAGTTGAACGCGGATGGCAGCAAAAGTGTTACTTTTGAGAGCGGCAAAAAGATGGACTTTGATCTTGTCATGATGGCGATTGGCCGTTCTCCCCGAACCAAGGATTTACAGCTGCAAAACGCCGGTGTCATGATCAAGAACGGTGGTGTGCAGGTGGACGAGTACTCGCGCACGAATGTTTCCAACATTTACGCCATCGGTGACGTCACAAATCGTGTCATGTTGACACCCGTGGCCATAAATGAAGCCGCTGCCCTTGTGGATACCATCTTTGGTACCACTCCGCGAAAGACGGACCACACCCGTGTGGCGAGTGCCGTCTTCTCTATTCCTCCAATTGGTACCTGCGGTCTCATTGAAGAGGTTGCATCCAAGCGCTACGAGGTGGTG

**5. Trypanothione synthetase (TcTrS)**

**Reference Gene: TcCLB.509099.50**

**>Scaffold AQP**

AGGCAACAAATGACGACTCTACAGTCTTTGGCGGTACCATTTGGCTGTGTGCAAGGCTATGCCCCTGGCGGTATTCCTGCGTACAGTAACAAGCATGAATCGTACTTTTCTGGGGAGCGAAGCATTGATGGAAATTTGTTTTGTGGGTTCAAGTACCAGTGCGTGGAGTTTGCGCGCCGATGGTTGTTTGAGCGTAAGTCTTTGGTTCTTCCCGATGTTGACTGGGCTGTGCACATCTTTAATTTAAAGGAGGTTTCGGATGCGCGCACGGGACAACCTGTTCGTTGCGTGGCGATTCGTAATGGTACCGCGGCGAAACCCGTGGTCGACTCGCTTCTTATTTACCCTTCCGACGATTATAGCCCAGTGGGCCATGTCGCTGCCATTACGGAGGTTGGGGACAAGTGGGTGCGCATTGCCGATCAAAACCATCGGTTTCACAAGTGGGATGCTAATTATGCGGCAGAGCTGCCGCTGATTCACGAGAAGGGCGTCTGGACGATTTTGGATCCGTTGGAGGATGAAGTGCTGAAGCCTCTCGGGTGGGTGACTTTCCCGGAGACACCCGATCGCAACCCGAATGAGCCGCTGGTTCTACACGAGTCGCTTCACTTCAAACGGGGGGAAGTGCCAACGCTGCGTCGTCTGACATTTACACCAAATTCGCGGGAAAAGGATTGGCTGGATCTCACGAATGAAGCCGAGGCGTACTTCGCGGGTGTCTGTGGTATCGATGTGAAGAATCCGAAAGTGGAAAAAGCCAGTTACTATCAGATGAACCGGGAGCTTTATTTGGACTGCGTCAAATACGGCAATCAACTTCATCAAATGTTTTTGGAGGCCACCAAGTTCGTCATTGGAAGTGACGAACAGCTAAGACTTTTTCACATTCCAGAGGAGTACTGGCCGCGTCTTCGATATTCGTGGGAGACACAACCGCATGCCATCACGGGACGCTTTGATTTTGCCTTTGACGAGGATACGCAGCAATTTAAGTGCTTTGAGTACAATGCTGATAGTGCCTCAACACTGCTGGAGTGCGGCGTTATTCAACAAAAGTGGGCGAGATCGGTTGGTCTCGATGACGGGACGACGTACAGTTCTGGGAGTTTGATTTGCCTCACGACTGCAATTGGCATGGAAATGGCAGGTAGTCACCGGACGTGTACACTTCCTTATCGACAAGGATGATGAGGAGTATTACACCGCCTTGTATGTTATGCAGCACGCTTCTGCGGCAGGATTGGAGACAAAA

**> TcCLB.509099.50**

ATGCCAACTCTACAGTCTTTGGCGGTACCATTTGGCTGTGTGCAAGGCTATGCCCCTGGCGGTATTCCTGCGTACAGTAACAAGCATGAATCGTACTTTTCTGGGGAGCGAAGCATTGATGGAAATTTGTTTTGTGGGTTCAAGTACCAGTGCGTGGAGTTTGCGCGCCGATGGTTGTTTGAGCGTAAGTCTTTGGTTCTTCCTGATGTTGAATGGGCTGTTCACATTTTTAATTTAAAGGAGGTTTCGGATGCGCGCACGGGACAAAATGTTCGTTGCGTGGCGATTCGTAATGGTACCGCGGCGAAACCCGTGGCCGACTCGCTTCTTATTTACCCTTCCGACGATTATAGCCCAGTGGGCCATGTCGCTGCCATTACGGAGGTTGGGGACAACTGGGTGCGCATTGCCGATCAAAACCATCGGTTTCACAAGTGGGATGCCAATTATGCGGCGGAGCTGCCGCTGATTCACGAGAAGGGCGTCTGGACGATTTTGGATCCGTTGGAGGATGAAGTGTTGAGGCCTCTCGGGTGGGTGACGTTTCCGGAGACACCCGATCGCAACCCGAATGAGCCGCTGGTTCTACACGAGTCGCTTCACTTCAAACGGGGGGAAGTGCCAACGCTGCGTCGTCTGACATTTACACCAAATTCGCGGGAAAAGGATTGGCTGGATCTCACGAATGAAGCCGAGGCGTACTTCGCAAGTGTCTGTGGTATTGATGTGAAGAATCCGAAAGTGGAAAAAGCCAGTTACTATCAGATGAACCGGGAACTTTATTTGGACTGCACCAAATACGGCAATCAACTTCATCAAATGTTTTTGGAGGCCACCAAGTTCGTTATTGGAAGTGACGAACAGCTAAGGGTTTTTCGTATTCCAGAGGAGTACTGGCCGCGTCTCCGACATTCATGGGAGACACAACCGCATGCCATCACGGGACGCTTTGATTTTGCCTTTGATGAGGATACGCAGCAATTTAAGTGCTTTGAGTACAATGCTGATAGTGCCTCAACACTGCTGGAGTGCGGAGTTATTCAACAAAAATGGGCGAGATCGGTTGGTCTCGATGACGGGACGACGTACAGCTGTGGGAGTTTGATTTCCTCACGACTGCAATTGGCATGGGAAATGGCAGAAGTCACCGGGCGTGTACACTTCCTTATCGACAAGGATGATGAGGAGTATTACACCGCCTTGTATGTTATGCAGCACGCTTCTGCGGCAGGATTGGAGACAAAACTTTGCGTTCTTTTCGATGAATTTCATTTTGATGAGAATGGCGTCGTTGTGGACTCCGATGGCGTCGCCGTCACAACGGTGTGGAAGACATGGATGTGGGAAACTGCCATTGCGGATCATCAGAAGGCCCGGGTGCAGCGCGGGAATGATTGGCGGCCAACGCCAAAGGATGAGGTTCGGCTTTGTGACATTCTTCTTGGGCCGAACTGGGACTTGCGTGTGTTTGAGCCCATGTGGAAAATTATTCCCAGCAACAAGGCTATCCTGCCCATTATTTACAACAAGCACCCAGACCATCCAGCGTTGCTGCGGGCCAGCTATGAACTCACGGTTGAGCTGCAGCGCACCGGTTACGTGAAGAAGCCCATCGTTGGTCGTGTCGGTCGAAATGTCACCGTCACAGAGGCGTCTGGTGACATTGCCGCGAAATCGGATGGAAATTTTTCGGACCGCGACATGGTCTACCAAGAGCTTTTTCGCCTGCCCGAGAGGGATGGCTACTACGCCATACTCGGCGGCTGGGTGATTGGCGATGTCTACTGCGGCACTGGTGTCCGCGAGGACAAGACAATCATTACGGGACTCGAGTCACCATTCATCGCCCTTCGAGTGTATCAGGGCGCCCCACGACGTCCACTGACCCACGAAGATCTCGACAAGGCGGAAGCCGCGGCCGTCGGTGGTGGCTTGAAAACGTGA

**6. Tryparedoxin peroxidase mitochondrial (TcMPX)**

**Reference gene: TcCLB.509499.14**

**>Scaffold AQP**

AAACACACAAGAAAAGCTTTGTCATGTTTCGTCGTATGGCCGTGACTTCGTTGCAGAAGGGTCTTTCACGCCGAGCTTTCTGCAACACCCTGCGACTTCTTAACCTGGACTACCAAGCATATAAGACTGCCACGGTGCGCGAGGCAGCACCTGAGTGGGCAGGAAAGGCTGTCGTGAATGGAAAAATCCAGGACATCAGCTTGAATGACTACAAGGGCAAGTATGTTGTTCTTCTTTTTTATCCTATGGATTTTACCTTTGTGTGCCCCACAGAAATCACAGCCTTTTCAGATGCTCAGGCAGAATTTGATAAGATTAACACCCAGGTCGTGGCTGTATCATGCGACAGCCAATATTCACACCTTGCTTGGATAAACACTCCAAGAAATAAAGGTGGCCTTGGGGAAATGAGTATTCCTGTGCTTTCTGATCTCACCAAGGAAATTGCCCGCGACTATGGAGTTTTGATCGAGGAGCAAGGGATCTCTCTGCGCGGACTGTTCATCATTGACGATAAAGGGATTCTGCGCCACATCACGGTGAATGACTTGCCAGTGGGCAGAAATGTTGAAGAGGTGCTTCGGGTTGTGCAGGCGTTTCAGTATGTGGACAAAAACGGAGATGTAATTCCATGTAACTGGAGGCCAGGCAAACCGACAATGAAAACTGAGAAGGCAAATGAATATTTTGAGAAAACGCATGAGTTCAGATGGTAGAAGGAGCATTGATGCATG

**>TcCLB.509499.14**

ATGTTTCGTCGTATGGCCGTGACTTCGTTGCAGAAGGGTCTTTCACGCCGAGCTTTCTGCAACACCCCGCGACTTCTTAACCTGGACTACCAAGCATATAAGACTGCCACGGTGCGCGAGGCAGCACCTGAGTGGGCAGGAAAGGCTGTCGTGAATGGAAAAATCCAGGACATCAGCTTGAATGACTACAAGGGCAAGTATGTTGTTCTTCTTTTTTATCCTATGGATTTTACCTTTGTGTGCCCCACGGAAATCACAGCCTTTTCAGATGCTCAGGCTGAATTTGATAAGATTAACACCCAGGTCGTGGCTGTATCGTGCGACAGCCAATATTCACACCTTGCTTGGATAAACACTCCAAGAAATAAAGGTGGCCTTGGGGAAATGAGTATTCCTGTGCTTTCTGATCTCACCAAGGAAATTGCCCGCGACTATGGAGTTTTGATCGAGGAGCAAGGGATCTCTCTGCGCGGACTGTTCATCATTGACGATAAAGGGATTCTGCGCCACATCACGGTGAATGACTTACCAGTGGGCAGAAATGTTGAAGAGGTGCTTCGGGTTGTGCAGGCGTTTCAGTATGTGGACAAAAACGGAGATGTAATTCCATGTAACTGGAGGCCAGGCAAACCGACAATGAAAACTGAGAAGGCAAATGAATATTTTGAGAAAAACGCATAA

**7. Tryparedoxin peroxidase cytosolic (TcCPX)**

**Reference gene: TcCLB.487507.10**

**>Scaffold AQP**

CACAATAAGCAACACAAAAAACAGCCTTTTATTTTCCAAGACAATGTCCTGCGGAGACGCAAAGCTCAACCACCCGGCGCCCGACTTCAATGAGACGGCGCTGATGCCCAACGGCACCTTCAAGAAGGTGGCTCTCAGCTCCTACAAGGGTAAGTGGCTGGTGCTCTTCTTCTACCCGATGGACTTCACCTTCGTCTGCCCCACAGAGATCTGCCAATTCTCGGACCGTGTGAAGGAGTTCTCTGACATTGGCTGCGAGGTGCTTGCCTGCTCCATGGACAGCGAGTACTCCCATCTGGCCTGGACAAGCGTTGAGCGCAAGCGTGGCGGACTTGGCCAGATGAACATCCCCATTCTTGCCGACAAGACCAAGTGCATCATGAAGTCGTATGGTGTGCTGAAGGAGGAGGATGGCGTGGCGTACCGTGGCCTTTTCATCATCGACCCGAAGCAGAACCTGCGGCAGATCACCGTCAACGACCTCCCCGTTGGCCGCGACGTGGACGAGGCCCTTCGCCTTGTGAAGGCGTTCCAGTTTGTGGAGAAGCATGGCGAGGTGTGCCCCGCCAACTGGAAGCCCGGTGACAAGACGGATGAAGCCGGATCCCGAAAAGTCAAGGAGTACTTGGTGCTGTCGCGTAGCGTGGCGGTTCACAGTCTCATTGTTGACATCTTGTTTTTTTTTCGTTTTTTAGTTTTTTTCGTTTTTTGCCTTTTGATGTACACGTTTTTTACTTTTGGTTTTTTTAAGTTTTTCGTTGGGGG

**> TcCLB.487507.10**

ATGTCCTGCGGAGACGCAAAGCTCAACCACCCGGCGCCCGACTTCAATGAGACGGCGCTGATGCCCAACGGCACCTTCAAGAAGGTGGCTCTCACCTCCTACAAGGGCAAGTGGCTGGTGCTCTTCTTCTACCCGATGGACTTCACCTTCGTCTGCCCCACAGAGATCTGCCAATTCTCGGACCGTGTGAAGGAGTTCTCTGACATTGGCTGCGAGGTGCTTGCCTGCTCCATGGACAGCGAGTACTCCCATCTGGCCTGGACAAGCATTGAGCGCAAGCGTGGCGGACTTGGCCAGATGAACATCCCCATTCTTGCCGACAAGACCAAGTGCATCATGAAGTCGTATGGTGTGCTGAAGGAGGAGGATGGCGTGGCGTACCGTGGCCTTTTCATCATCGACCCGAAGCAGAACCTGCGGCAGATCACCGTCAACGACCTCCCCGTTGGCCGCGACGTGGACGAGGCCCTTCGCCTTGTGAAGGCGTTCCAGTTCGTGGAGAAGCATGGCGAGGTGTGCCCCGCCAACTGGAAGCCCGGTGACAAGACGATGAAGCCGGATCCCGAAAAGTCCAAGGAGTACTTTGGTGCTGTCGCGTAG
